# De Novo Expressed Vpr Stimulates HIV-1 Replication in T Cells

**DOI:** 10.3390/v17070958

**Published:** 2025-07-07

**Authors:** Blessing Enya, Jacek Skowronski

**Affiliations:** Department of Molecular Biology and Microbiology, Case Western Reserve School of Medicine, 10900 Euclid Ave., Cleveland, OH 44106, USA; blessing.enya@case.edu

**Keywords:** HIV-1 Vpr, accessory proteins, T cells, viral replication, virion-associated Vpr, preintegration silencing, retroviral pathogenesis

## Abstract

Vpr, a virion-associated accessory virulence factor of HIV-1, promotes virus replication in both T cells and macrophages. Although Vpr’s early activity—antagonism of preintegration silencing and host restriction factors—has been documented, the relative contribution of virion-associated versus de novo expressed Vpr to HIV-1 replication fitness remains unclear. Here, we developed a T cell-based system that genetically separates early and late Vpr functions by combining tetracycline-inducible Vpr expression in CEM.SS T cells with vpr-deficient HIV-1 constructs and Gag p6 mutations that block Vpr packaging. CEM.SS T cells have been shown to recapitulate the positive effect of Vpr on HIV-1 replication observed in activated primary T cells. Using pairwise replication fitness assays under spreading infection conditions, we demonstrate that de novo synthesized Vpr exerts the dominant effect on HIV-1 replication in T cells, while virion-associated Vpr plays a lesser role. Somewhat unexpectedly, our findings reveal that antagonism of preintegration HIV-1 silencing by virion-associated Vpr is unlikely to be the major driver of enhanced HIV-1 replication in proliferating T cells. Instead, this function may play a more prominent role in the infection of non-dividing T cells and/or other cell types.

## 1. Introduction

Accessory virulence factors of DNA and RNA viruses are generally dispensable for viral replication ex vivo in established cell lines; however, they are critical for efficient replication, dissemination, and survival within natural hosts [1,2,3,4]. These proteins enhance viral fitness by antagonizing innate and adaptive immune responses and/or by augmenting viral gene expression at the transcriptional or post-transcriptional level. They may act at various stages of the viral replication cycle—either early or late—and can influence complex biological processes, such as cell type tropism or transitions between latent and lytic infection phases. Herpesviruses provide a well-studied example, encoding a broad array of accessory proteins that modulate virus–host cell interactions throughout infection [5]. Notably, some of these proteins are specifically incorporated into virions, allowing them to exert their effects immediately upon their entry into the target cell, even before de novo viral gene expression begins. A classic example is the Herpesvirus VP16 protein, a transcriptional activator of viral immediate-early genes, which is delivered into the target cell as part of the viral tegument [6]. In general, the presence of an accessory protein within the infecting virion implies a functional role during early post-entry steps, prior to the activation of viral gene expression.

HIV-1 encodes four accessory proteins—Vif, Vpr, Vpu, and Nef—that counteract host restriction factors in CD4+ T cells and myeloid cells, the principal targets of HIV-1 infection [7,8,9]. Of these, Vif and Vpu are not packaged into virion and function primarily after the integration of the provirus, during late stages of the replication cycle. Vif targets APOBEC3 cytidine deaminases for proteasomal degradation by hijacking the CRL5 E3 ubiquitin ligase, thereby preventing lethal hypermutation of the viral genome during reverse transcription [10]. Vpu promotes CD4 degradation by engaging the CRL1/β-TrCP complex and counteracts the restriction factor Tetherin/BST-2. Through these actions, Vpu prevents the retention of HIV-1 Env in the ER and antagonizes Tetherin-mediated inhibition of virion release from the plasma membrane, thereby facilitating virion release [11,12]. Nef is packaged into HIV-1 virions [13,14,15], but no function has been attributed to virion associated Nef [13,14,15]. Nef reroutes CD4 and MHC-I molecules from recycling pathways toward lysosomal degradation by engaging clathrin adaptor complexes AP-1 and AP-2 [11,16]. Downregulation of MHC-I helps infected cells evade immune detection [17]. Nef also enhances HIV-1 infectivity by preventing the incorporation of the SERINC3 and SERINC5 restriction factors into budding virions, which would otherwise lead to an impairment of membrane fusion between HIV-1 and a target cell [11,18,19].

In contrast, Vpr is selectively incorporated into HIV-1 virions through its interaction with the p6 domain of the Gag polyprotein [20,21,22,23], a region also critical for virus budding and release [24]. Vpr enhances HIV-1 replication in both T cells and myeloid cells [25,26,27,28,29]. In activated primary CD4+ T cells and T cell lines such as CEM.SS and HPB.ALL, Vpr confers a substantial replication advantage that can be quantified using sensitive pairwise replication fitness assays [30].

The packaging of Vpr in HIV-1 virions suggests a role at the early post-entry stages of the replication cycle. Interestingly, virion-associated Vpr has been shown to antagonize epigenetic silencing of unintegrated HIV-1 DNA [31,32]. Epigenetic silencing of the incoming retroviral genome before provirus integration is a conserved host antiviral defense mechanism [33,34]. Vpr also counteracts restriction of HIV-1 infection by the DNA repair enzyme HLTF, which similarly acts prior to provirus integration [29,30]. Beyond these early effects, Vpr has been reported to enhance transcription directed by HIV-1 regulatory elements to enhance viral gene expression, an effect that is observable in the context of integrated proviruses [26,35,36,37]. However, the relative contribution of virion-associated versus de novo expressed Vpr to HIV-1 replication in T cells remains poorly understood.

Here, we describe a novel T cell-based experimental system to genetically separate and quantify the effects of Vpr exerted early (via virion delivery) and late (via de novo synthesis) during the HIV-1 replication cycle. Briefly, we engineered replication-competent HIV-1 clones lacking vpr and expressing the tetracycline-controlled transactivator (tTA), which allows inducible gene expression directed by tetracycline response element (TRE) containing promoters [38], and introduced tetracycline inducible vpr transgenes into CEM.SS T cells. We further disrupted Vpr packaging by mutating the Gag p6 domain [39], enabling a clean genetic separation of early and late Vpr effects. Using pairwise replication fitness assays in the context of spreading infection, we show that the late-stage activity of de novo expressed Vpr plays the predominant role in enhancing HIV-1 replication in T cells, relative to the contribution of virion-associated Vpr.

## 2. Materials and Methods

### 2.1. HIV-1 Proviral Clones

Replication competent HIV-1 NL4-3 derived proviral constructs carrying a red fluorescent protein gene (RFP/mRFP) and a functional vpr gene (HIV-1.R+), or an inactivated vpr gene with the glutamine Q8 codon substituted with a stop codon (CAA > TAA; HIV-1.R-) were previously described as HIV-1.mRFP.vpr.wt and HIV-1.RFP.vpr.Q8* [30]. The viruses are isogenic, except for a mutation in vpr and an array of silent mutations in the mRFP gene. The wild-type and mutated RFP sequences provide unique sites for primer annealing that allow their quantification by qPCR in coinfection assays [30].

The HIV-1.R-.mRFP.tTA.wt proviral clone harbors a cassette encoding a polyprotein comprising the mRFP fused to a self-cleaving peptide (2A) spacer connecting to the tetracycline-dependent transcriptional activator protein (tTA) [38,40], followed by an IRES element directing the translation of the Nef protein. The HIV-1.R-.mRFP.tTA.wt proviral clone was constructed by replacing the HIV-1.mRFP.vpr.wt EagI-XhoI fragment with the EagI-XhoI fragment comprising the above described mRFP-2A-tTA.wt-IRES-nef cassette, using the NEBuilder HiFi DNA Assembly Cloning kit (New England Biolabs, Ipswich, MA, USA). The nucleotide sequence and peptides encoded by this cassette are shown in Supplementary Figure S1.

The HIV-1.R-.RFP.tTA.** is isogenic except that it harbors a wild-type RFP gene, and the tTA gene is inactivated by substituting the serine S3, arginine R4, and glutamine Q77 codons with termination codons (tTA.S3* [TCA > TGA], tTA.R4* [AGA > TGA]), and tTA.Q77 [CAA > TA]), the latter resulting in a frameshift mutation generating a TAG stop codon (Supplementary Figure S1).

The HIV-1.p6.4A.R-.mRFP.tTA.wt proviral clone is isogenic to HIV-1.R-.mRFP.tTA.wt, except for the substitution of Gag p6 residues 15–18 (FRFG) with alanine (AAAA) [39]. Site-directed mutagenesis was performed using the QuikChange Lightning kit (Agilent, Santa Clara, CA, USA). All mutations and constructs were verified by whole plasmid sequencing (Genewiz, South Plainfield, NJ, USA).

### 2.2. Retroviral Expression Vectors

The pRetroX-TRE3G vector was obtained from Clontech (Mountain View, CA, USA). The pcDNA3.1 vector encoding HA- or FLAG- epitope-tagged codon-optimized HIV-1 NL4-3 vpr gene was provided by Dr. Jinwoo Ahn (U. Pittsburgh, Pittsburgh, PA, USA). The FLAG-tagged codon-optimized HIV-1 NL4-3 vpr gene and GFP gene, derived from the TRIP retroviral vector [41], were inserted between the BamHI and EcoRI sites in the RetroX-TRE3G vector, using the NEBuilder HiFi DNA Assembly Cloning kit (New England Biolabs).

### 2.3. Cells Lines

HEK293T cells were maintained in DMEM medium (Cytiva Life Sciences, Marlborough, MA, USA) supplemented with 10% (vol/vol) FBS (Hyclone, Wilmington, DE, USA), and 1% (vol/vol) 100× penicillin-streptomycin-L-glutamine (Corning, Corning, NY, USA). CEM.SS T cells were obtained from the NIH AIDS Reagent Program [42,43,44]. CEM.SS T cells harboring FLAG-tagged doxycycline-inducible codon-optimized HIV-1 NL4-3 vpr or GFP transgenes, or the control empty vector, were constructed by transducing the cells with the respective RetroX-TRE3G vector and then maintained in RPMI 1640 medium supplemented with 10% (vol/vol) Tet System Approved FBS (Clontech), 1% (vol/vol) 100× penicillin-streptomycin-L-glutamine, and puromycin (1 μg/mL).

### 2.4. Virus Production in HEK293T Cells

HEK293T cells [45] were transfected with the HIV-1 or RetroX-TRE3G proviral clone using the calcium phos-phate co-precipitation method, as previously described [46]. Then, 24 h after transfection, the cell culture supernatant was collected and centrifuged at 3000 rpm for 10 min at room temperature to pellet cell debris. The supernatant containing HIV-1 particles was treated with 40 units/mL DNase I (MilliporeSigma, Burlington, MA, USA) for 1 h at 27 °C, sterile filtered (MilliporeSigma), and the virus concentrated by pelleting through a 20% sucrose cushion, as previously described [47]. The supernatant containing RetroX-TRE3G virus was filtered through a 0.45 μm filter (Fisher Scientific, Hampton, NH, USA) and concentrated using a RetroX concentrator (Takara, San Jose, CA, USA). The pelleted virus was resuspended in TNE buffer [48], and virus titer was determined based on infectivity to CEM.SS T cells as revealed by RFP fluorescence. Cells were harvested, 48 h post infection (hpi), and fixed with 0.4% formaldehyde, and RFP-positive cells displaying red fluorescence were quantified using a (BD LSRFortessa flow cytometer. The collected data were analyzed using FlowJo software v10.10 (Ashland, OR, USA).

### 2.5. Production of HIV-1.R Virions Trans-Complemented with Vpr in HEK293T Cells

HIV-1.R-.mRFP.tTA.wt or HIV-1.R-.RFP.tTA.** proviral clones harboring vpr with the inactivating Q8* mutation were transiently co-transfected with pcDNA3.1 vector expressing FLAG epitope-tagged codon-optimized HIV-1 NL4-3 Vpr, into HEK293T cells, by the calcium phosphate co-precipitation method. Cell culture supernatant was collected, processed, and viral particles concentrated, as described in the section above.

### 2.6. Virus Production in CEM.SS T Cells

CEM.SS-TRE3G and CEM.SS-TRE3G-vpr T cells (10^5^ cells/mL, 10 mL) were infected with HIV-1.R-.mRFP.tTA.wt or HIV-1.R-.RFP.tTA.** expressing either p6.wt or p6.4A at multiplicity of infection (moi) of 3. Infection was terminated after 24 h by pelleting the cells at 1500 rpm for 5 min, and the residual cell-free virus was removed by suspending the cell pellets in PBS and pelleting again, three times. The final cell pellets were suspended in complete RPMI medium supplemented with puromycin (1 μg/mL) and doxycycline (100 ng/mL). In total, 1 to 2 milliliters of cell culture supernatant were collected immediately following the last wash and at 24 h intervals until the 96 h timepoint, and titrated. The viral supernatant collected at the 96 h timepoint was concentrated with RetroX (Takara), the pelleted viruses resuspended in TNE buffer [48], and virus titers were determined based on infectivity to CEM.SS T cells.

### 2.7. Pairwise Replication Competition Assay (PRCA)

PRCA was performed essentially as previously described [30]. In brief, the competing viruses were first normalized based on their infectivity to CEM.SS T cells in single-cycle replication assay. Single-cycle replication assay was performed by infecting CEM.SS T cell cultures with each virus, supplementing the cell culture medium 20 hpi with 10 μg/mL AMD3100 Bicyclam entry inhibitor (NIH AIDS Reagent Program, ATCC, Manassas, VA, USA) to prevent virus spread, and quantifying the number of RFP-positive cells 48 hpi with a BD LSRFortessa flow cytometer. Subsequently, the viruses were mixed at an approximate 1:1 ratio, treated with 2 units of Turbo DNase (ThermoFisher, Waltham, MA, USA) in 50 µL RPMI supplemented with 1× Turbo DNase buffer at 37 °C for 20 min, RNA was extracted using RNeasy kit (Qiagen, Germantown, MD, USA), reverse transcribed, and RFP and mRFP amplicons were quantified by RT-qPCR [30]. Based on the qPCR data, virus pairs were mixed at the adjusted 1:1 ratio and used to infect 10^5^ CEM.SS-TRE3G T cells or CEM.SS-TRE3G-Vpr T cells, at an moi between 0.006 and 0.02, in 1 mL of complete RPMI1640 medium supplemented with 100 ng/mL doxycycline. At 3-, 5-, and 7- days post infection (dpi), the percentage of RFP-positive cells was determined using BD LSRFortessa flow cytometer, and the infected cultures with fewer than 5% RFP-positive cells at 7 dpi were selected for quantification of the competing viruses by quantitative real time PCR (qPCR). The 5% cutoff served to minimize the frequency of cells co-infected with both competing viruses, which can lead to recombination events. Infected cell cultures were collected into 1.5 mL maximum recovery tubes (Axygen, Seattle, WA, USA) and pelleted by centrifugation at 500 rpm for 5 min. DNA was isolated using the DNeasy Blood and Tissue Kit (Qiagen), and DNA concentrations were quantified using a Thermo Scientific Nanodrop 2000 Spectrophotometer. The DNA was then diluted to 10 ng/μL for qPCR quantification of cell associated viral DNA. qPCR was performed on a StepOnePlus Real-Time PCR machine. Setups and standards for the quantification of mRFP and RFP amplicons were previously described [30]. Three technical replicates were analyzed for each DNA sample.

### 2.8. Cell and Virus Lysates, and Immunoblotting

Cells were collected 24 h post transfection, pelleted by centrifugation at 1500 rpm for 5 min, washed with PBS, and lysed on ice with a lysis buffer (50 mM Tris-HCl pH 7.5, 150 mM NaCl, 1% Triton X-100, and 10% glycerol) supplemented with a protease inhibitor cocktail (MilliporeSigma). After 30 min of incubation, lysates were spun down at 14,000 rpm for 15 min at 4 °C, and the protein concentration in the supernatants was determined using the Pierce BCA Protein Assay Kit (ThermoFisher). Aliquots of extracts containing 30 μg of protein, or aliquots of concentrated virus preparations, were supplemented with Laemmli sample buffer (250 mM Tris-HCl pH 6.8, 10% SDS, 30% glycerol, 5% β-mercaptoethanol, 0.02% bromophenol blue) and denatured for 20 min at 95 °C. The denatured samples were separated by SDS-PAGE, transferred to a PVDF Immobilon-FL membrane (MilliporeSigma), immunoblotted with appropriate primary antibodies, and immune complexes revealed with fluorescent secondary antibodies (KPL) and imaged using an Odyssey Infrared Imager (Licor, Lincoln, NE, USA), as previously described [48]. The following primary antibodies were used: rabbit anti-Vpr polyclonal (ARP-3537; BEI Resources), mouse anti-FLAG M2 mAb (MilliporeSigma), mouse anti-Splicing Factor 2 mAb (SF2, provided by A. Krainer, Cold Spring Harbor Laboratory, Cold Spring Harbor, NY, USA), mouse anti-HA mAb (12CA5) [49], and mouse anti-HIV-1 CA mAb (183-H12-5c) primary antibodies [50]. The 12CA5 mAb and 183-H12-5c mAb were produced inhouse as previously described [48]. The following secondary antibodies were used (KPL): IRDye^®^ 680RD Goat anti-Mouse IgG (926-68070), IRDye^®^ 800CW Goat anti-Mouse IgG (926-32210), IRDye^®^ 680RD Goat anti-Rabbit IgG (926-68071) and IRDye^®^ 800CW Goat anti-Rabbit IgG (926-32211). Image blots were collected using an Odyssey infrared imager (Licor) and quantified with the Image Studio software v. 6.0.0.28 (Licor).

### 2.9. Expression from Unintegrated HIV-1

CEM.SS T cells (10^5^ cells in 1 mL) cultured in the presence of 0.1 μg/mL integrase inhibitor Raltegravir were infected with HIV-1 at moi of 0.3, 1, and 3. Alternatively, experiments were performed with HIV-1 carrying the inactivating D116N substitution in Integrase, in absence of Raltegravir. At 48 hpi, the cells were fixed with 0.4% paraformaldehyde, analyzed by flow cytometry (BD LSRFortessa), and the data analyzed using FlowJo software (FlowJo LLC, Ashland, OR, USA).

### 2.10. Statistical Analysis

Statistical significance of the data was analyzed using one-way ANOVA with post hoc Tukey test, or *t*-test. *p*-values less than 0.05 were considered statistically significant.

### 2.11. Reagents Obtained Through the NIH HIV Reagent Program, Division of AIDS, NIAID, NIH

CEM-SS T cells, ARP-776, contributed by Dr. Peter L. Nara [30,39,44,48], Anti-Human Immunodeficiency Virus 1 (HIV-1) p24 Hybridoma (183-H12-5C), ARP-1513, contributed by Dr. Bruce Chesebro and Dr. Hardy Chen [50], Bicyclam JM-2987 (hydrobromide salt of AMD-3100), ARP-8128, contributed by DAIDS/NIAID, polyclonal Anti-Human Immunodeficiency Virus Type 1 Vpr Protein, Residues 1 to 50 (antiserum, Rabbit), ARP-11836, contributed by Dr. Jeffrey Kopp.

## 3. Results

### 3.1. Inducible Expression System for Trans-Complementation of HIV-1 Particles Produced from a vpr-Defective Provirus with HIV-1 Vpr

We previously demonstrated that HIV-1 Vpr enhances viral replication in primary T cells and model T cell lines, such as CEM.SS T cells, using a sensitive pairwise replication competition assay (PRCA; [30]). Our initial experiments revealed the presence of an early post-entry component contributing to this effect [30]. However, evidence from other laboratories, using different experimental systems, has supported both pre-integration and late-stage replication cycle functions of Vpr [25,26,27,30,37,51,52]. In this study, we further dissect the putative early and late replication effects of Vpr by genetically separating these functions.

As a first step, we developed a bipartite inducible expression system that enables trans-complementation of HIV-1 particles produced from a vpr-defective provirus with HIV-1 Vpr, which is selectively expressed in the infected CEM.SS T cells following provirus integration. This system combines a modified tetracycline-controlled transactivator (tTA) inserted into a proviral clone with a CEM.SS T cell line engineered to express Vpr from a tetracycline response element (TRE)-driven cassette [38]. It is compatible with pairwise replication fitness assays, such as the PRCA [30].

**Figure 1:**
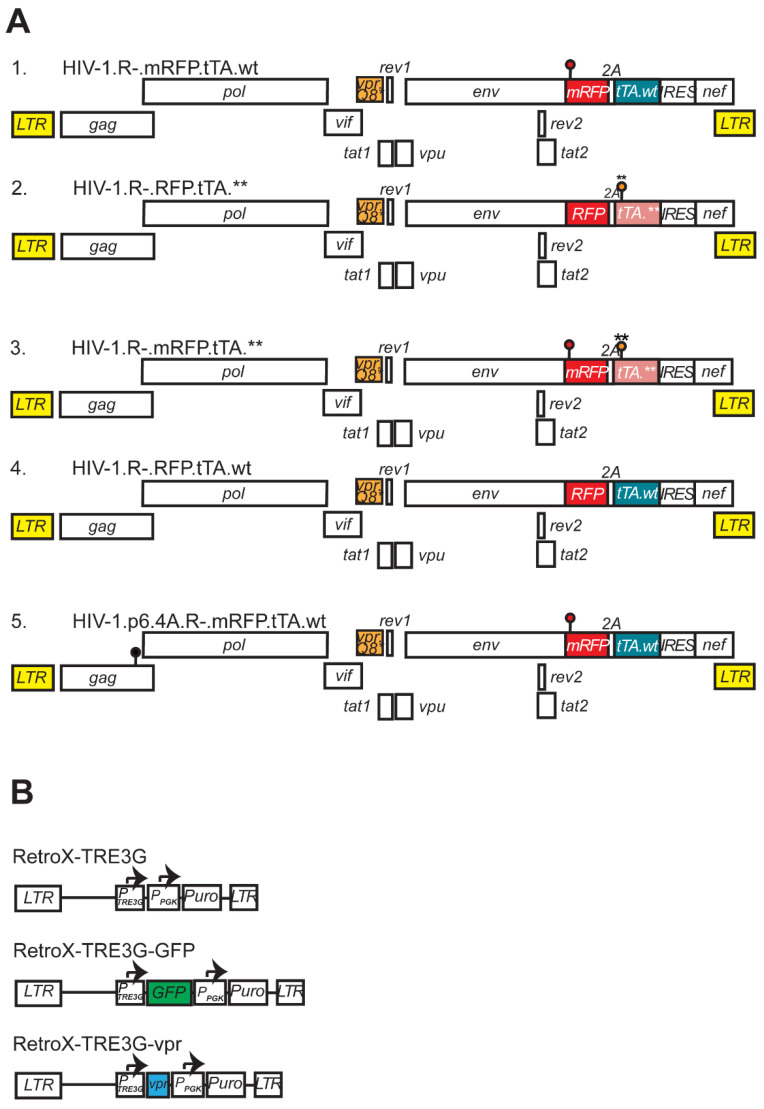
Schematic representation of proviral constructs. (**A**) Schematic of HIV-1 reporter constructs used in pairwise replication competition assays (PRCA). Long terminal repeats (LTR, highlighted in yellow), open reading frames encoding structural and accessory proteins, and the IRES element driving translation of the Nef protein are represented by rectangles. All viruses contain an inactivating mutation (Q8*) in the vpr gene (R–) and encode an RFP marker protein linked via a 2A self-cleaving peptide to a transactivator gene (tTA). The viruses are isogenic, differing only in their combinations of wild-type and mutated p6 (p6.wt, p6.4A), RFP (RFP/mRFP), and tTA (tTA.wt, tTA.**) genes. Mutations are denoted with lollipops: black for p6.4A, red for mRFP, and orange for tTA. Asterisks above the tTA lollipop denote premature termination codons. Sequence differences between RFP and mRFP allow quantification of each virus by qPCR in co-infection assays [30]. (**B**) Schematic of RetroX-TRE3G expression vectors. The RetroX-TRE3G-GFP and RetroX-TRE3G-vpr retroviral vectors carry a GFP or codon-optimized HIV-1 NL43 vpr gene under control of tetracycline-responsive promoter (PTRE3G). PPGK denotes the phosphoglycerate kinase gene promoter driving the puromycin resistance gene (Puro) expression.

We constructed a pair of reporter viruses based on previously described HIV-1 NL4-3-derived vpr-defective viruses, which were used to demonstrate the positive effect of Vpr on HIV-1 replication in T cells using PRCA [30]. As shown in Figure 1(A1,A2), in both reporter constructs, the vpr gene was inactivated by replacing the glutamine (Q8) codon with a termination codon (Q8*). Both viruses encoded a tripartite polyprotein comprising red fluorescent protein (RFP or mRFP, an A2 self-cleaving peptide, and the tTA transactivator) [38,40]. The two constructs were isogenic except for (i) a cluster of silent mutations in the RFP gene (mRFP), and (ii) a cluster of premature stop codons in the tTA gene in one of the viral constructs (tTA.**). The wild-type and mutant RFP (mRFP) sequences provided unique tags, enabling selective detection and quantification of each virus in co-infection experiments via qPCR (Figure 1A in [30]).

**Figure 2:**
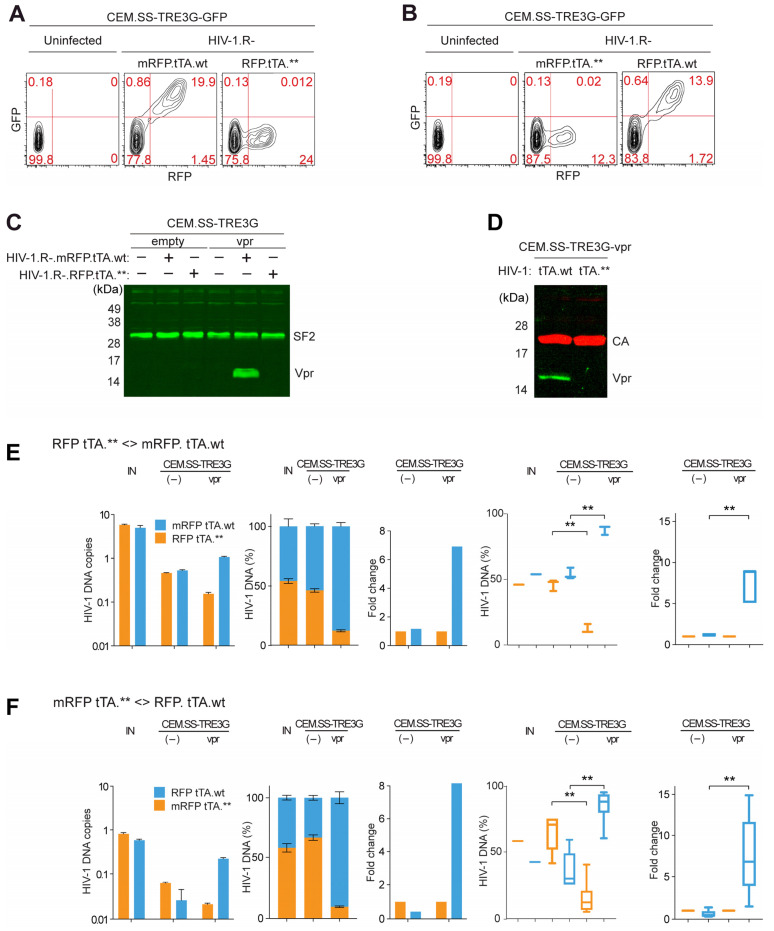
HIV-1 Vpr enhances HIV-1 replication when provided in trans in CEM.SS-TRE3G-vpr T cells. (**A**,**B**) HIV-1. R-.mRFP.tTA.wt induces GFP expression in CEM.SS-TRE3G-GFP T cells. Cells were infected with HIV-1.R-.mRFP.tTA.wt or HIV-1.R-.RFP.tTA.** (**A**) or with isogenic constructs where the tTA.wt and tTA.** genes were swapped (**B**) at an moi of 0.2 and cultured in the presence of 100 ng/mL doxycycline. GFP and RFP expression were quantified by flow cytometry at 3 dpi. Percentages of the GFP- and RFP-positive cells are indicated. Data are representative of three independent experiments. (**C**) HIV-1.R-.mRFP.tTA.wt induces Vpr expression in CEM.SS-TRE3G-vpr T cells. CEM.SS-TRE3G (empty) and CEM.SS-TRE3G-vpr (vpr) T cells were infected (+) or not (−), with either HIV-1.R-.mRFP.tTA.wt or HIV-1.R-.RFP.tTA.** at moi of 2, and cultured with 100 ng/mL doxycycline. Cell extracts prepared at 3 dpi were analyzed by immunoblotting for Vpr; Splicing Factor 2 (SF2) provided a loading control. Molecular weight standards are indicated on the left. The data shown are representative of three independent experiments. (**D**) Trans-complementation of HIV-1 particles produced from CEM.SS-TRE3G-vpr T cells with Vpr. Cells were infected with HIV-1.R-.mRFP.tTA.wt (tTA.wt) or HIV-1.R-.RFP.tTA.** (tTA.**) and viruses containing supernatants were collected 96 hpi. Viruses were concentrated, lysed, resolved by SDS-PAGE, and immunoblotted for CA and Vpr. Positions of CA and Vpr bands are indicated. E, F. Inducible Vpr expression enhances HIV-1 replication. (**E**) CEM.SS-TRE3G (–) and CEM.SS-TRE3G-vpr (vpr) T cells were infected with ~1:1 mixture of HIV-1.R-.mRFP.tTA.wt and HIV-1.R-.RFP.tTA.** at an moi of 0.01, cultured in the presence of 100 ng/mL doxycycline. At 7 dpi, DNA was extracted and qPCR performed to quantify cell-associated DNA for each competing virus using mRFP and RFP amplicons. Left panels—results from a representative experiment: (1) the number of copies of cell-associated HIV-1 DNA for each of the competing viruses. (2) relative percentages of cell-associated HIV-1 DNA of each of the competing viruses. (3) fold-change in functional (tTA.wt) over non-functional (tTA.**) virus. (IN): relative amounts of the competing viruses in the inoculum were determined by quantifying the RFP and mRFP amplicons in the reverse transcribed RNA isolated from the inoculum, using RT-qPCR. Right panels: compiled results from five independent experiments. Statistical significance (one-way ANOVA with post hoc Tukey test) is indicated above the panels. **—*p* < 0.01, ns—not significant. (**F**) Same as in panel E, but with tTA.wt and tTA. swapped between constructs.

The functionality of the reporter constructs was initially tested by transducing CEM.SS T cell populations harboring either the GFP (CEM.SS-TRE3G-GFP) or HIV-1 Vpr (CEM.SS-TRE3G-vpr) gene under the control of the TRE3G tetracycline response element, in the presence of doxycycline (Figure 1B). As shown in Figure 2A–C, transduction with HIV-1 carrying the intact, but not the defective, tTA gene (HIV-1.R-.mRFP.tTA.wt vs. HIV-1.R-.mRFP.tTA.**) resulted in the induction of GFP or Vpr expression in the target cells, as expected. Importantly, more than 98% of cells displaying RFP fluorescence—expressed from the proviral genome—were also positive for GFP fluorescence, indicating that tTA successfully induced transgene expression from a genomic locus in infected cells (Figure 2A). Identical results were obtained when the tTA.wt and tTA.** genes were swapped between the proviral reporter constructs (Figure 1A3, A4, and Figure 2B). Notably, tTA-induced Vpr was incorporated into HIV-1 virions released from the infected cells (Figure 2D). These observations demonstrate that the RFP/mRFP and tTA genes remained functionally linked; that the TRE3G-GFP transgenes were competent for transcriptional activation by tTA in all, or nearly all, infected cells; and that Vpr ectopically expressed in infected cells effectively trans-complemented HIV-1 particles produced from a vpr-defective provirus.

### 3.2. Vpr Provided in Trans in Infected CEM.SS T Cells Stimulates HIV-1 Replication

Next, using PRCA, we tested whether Vpr, induced by tTA from a chromosomal locus in HIV-1-infected target cells, can functionally replace Vpr expressed from its natural locus in the HIV-1 provirus. Initially, we sought to exclude the possibility that tTA expression alone could modulate HIV-1 replication in the absence of Vpr. As a negative control, CEM.SS-TRE3G cells harboring an empty TRE3G cassette were infected with an approximately 1:1 mixture of two viruses (Figure 2E), one harboring the functional tTA gene and the other a disrupted version. Infections were performed at moi of ~0.01 to minimize dual infection events, which could lead to recombination between the two competing viruses [30]. Seven days post-infection, cell-associated HIV-1 DNA was quantified by qPCR. As expected, both viruses replicated at similar rates, with their relative proportions remaining unchanged at 7 dpi. This result indicated that neither the tTA.wt nor the tTA.** gene modulates HIV-1 replication in the absence of Vpr in this experimental system.

In contrast, when CEM.SS T cells carrying the tTA-inducible TRE3G-Vpr cassette (CEM.SS-TRE3G-Vpr) were infected with the same virus mixture, the virus carrying the intact tTA.wt gene produced 7-fold more progeny than the one harboring the disrupted tTA coding sequence (tTA.**), under the exact same growth conditions. This enhancement was evident from a marked increase in the cell-associated DNA of the virus with the intact tTA.wt gene, which accounted for approximately 90% of the total cell-associated HIV-1 DNA at 7 dpi (Figure 2E). Similar results were observed in multiple biological replicate experiments (Figure 2E, two rightmost panels).

These findings were further corroborated using a complementary pair of HIV-1 reporter constructs in which the tTA.wt and tTA.**genes were swapped (Figure 2F). Together, these results demonstrate that Vpr, when ectopically expressed specifically in infected target T cells, can functionally trans-complement vpr-defective HIV-1. Importantly, the magnitude of the positive effect of Vpr observed in the trans-complementation setting described above is not markedly different from what we previously reported for Vpr expressed from its native context in the HIV-1 provirus [30].

### 3.3. Virion-Associated Vpr Is Not Critical for Vpr’s Positive Effect on HIV-1 Replication

**Figure 3:**
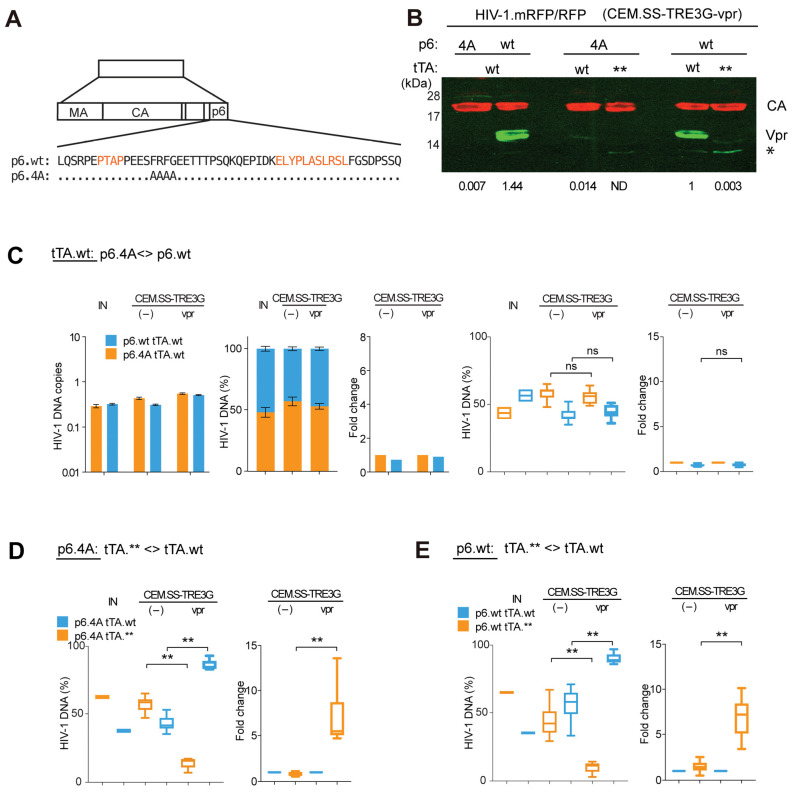
Virion-associated Vpr plays a minor role in enhancing HIV-1 replication. (**A**) Location of the 4A substitution in HIV-1 p6 amino acid sequence. The p6.4A sequence [39] is aligned with that of the HIV-1 NL43 p6 (p6.wt), in single letter code. Dots indicate identical amino acids. L-domain motifs are highlighted in orange. (**B**) p6.4A disrupts Vpr packaging into virions. CEM.SS-TRE3G-Vpr T cells were infected with the indicated HIV-1.R- reporter viruses in the presence of doxycycline. Supernatants collected 96 hpi which were processed for PAGE immunoblotting of CA and Vpr. tTA (tTA.wt, tTA.**) and p6 (p6.wt, p6.4A) genotypes of the viruses are noted. Positions of bands corresponding to CA and Vpr are indicated. Asterisk (*) marks a non-specific band cross-reactive with anti-Vpr antibody. Fluorescent signals were quantified with Odyssey Imager. Virion-associated Vpr levels, shown below the blot, were normalized to CA, then to a reference (p6.wt/tTA.wt) sample. The relative Vpr content of the virions, normalized to CA content and then to that in one of the HIV-1 with p6.wt and tTA.wt virus preparations, is shown below each lane. (*) indicates the position of a nonspecific band cross-reacting with the anti-Vpr antibody. (**C**) Impact of p6.4A on Vpr-mediated enhancement of replication. PRCA was performed using HIV-1 R- viruses with either p6.wt or p6.4A, both expressing tTA.wt. Experiments were performed and analyzed as described in the Figure 2C legend. Results from three independent experiments are compiled. Statistical significance (one-way ANOVA with post hoc Tukey test) is indicated above the C, D and E panels. **—*p* < 0.01, ns—not significant (**D**) PRCA with two p6.wt viruses: one expressing tTA.wt, the other tTA.**. Four independent replicates are compiled. (**E**) PRCA with two p6.4A viruses: one expressing tTA.wt, the other tTA.**. Four independent replicates are compiled.

Vpr is incorporated into the HIV-1 virion via the p6 component of Gag, whose L-domain function is essential for virus egress [20,21,23]. Previous studies have identified p6 mutations that specifically disrupt Vpr incorporation into virions without impairing late-domain function [39]. We took advantage of one such mutation, referred to here as 4A (p6.4A), illustrated in Figure 3A, to evaluate the contribution of virion-associated Vpr to HIV-1 replication in the CEM.SS T cell model. The p6.4A mutation was introduced into HIV-1 reporter proviral clones harboring the tTA gene (Figure 1A5). As shown in Figure 3B, the Vpr content of HIV-1 virions containing the mutated p6.4A peptide incorporated 200- to 300-fold less Vpr than control virions carrying wild-type p6 (p6.wt), with both types produced in CEM.SS-TRE3G-vpr cells.

We first asked whether the p6.4A mutation affects HIV-1 replication in control CEM.SS cells. To test this, we assessed the relative fitness of a pair of HIV-1 reporter viruses that differed only in the p6 locus and both encoded the functional tTA transactivator (tTA.wt). One virus, produced with wild-type p6 (p6.wt), efficiently packaged Vpr into the virion (HIV-1.p6wt.R-.RFP.tTA.wt). The other, incorporating the p6.4A variant, recruited minimal amount of Vpr into the virion (HIV-1.p6.4A.R-.mRFP.tTA.wt). CEM.SS-TRE3G T cells were infected with an approximately 1:1 mixture of the two viruses at low moi, and cell-associated DNA from each virus was quantified by qPCR at 7 dpi. As shown in Figure 3C, both viruses replicated at similar rates. The p6.4A variant showed a slight but reproducible fitness advantage in the absence of Vpr, although this difference was not statistically significant and was not pursued further.

Remarkably, when the same virus pair was tested in CEM.SS-TRE3G-vpr cells, their relative replication fitness remained identical—despite their virions containing vastly different amounts of Vpr. These results support a model in which the positive effect of Vpr on HIV-1 replication is primarily mediated by Vpr expressed in infected cells rather than by Vpr incorporated into the virion.

To corroborate and extend these findings, we evaluated another pair of HIV-1 reporter viruses, both containing the p6.4A mutation that abrogates Vpr packaging, but differing in their tTA coding sequences—one functional and one disrupted (Figure 3D). Notably, in CEM.SS-TRE3G-vpr cells, replication of the virus carrying the functional tTA gene was strongly stimulated. Importantly, the magnitude of stimulation (fold-change) was comparable to that observed for the corresponding isogenic pair of viruses harboring wild-type p6 instead of the p6.4A variant (Figure 3E).

Together, these observations demonstrate that the ability of Vpr to enhance HIV-1 replication in the CEM.SS T cell model is largely independent of its incorporation into the virion. This cleanly dissociates the functions of Vpr delivered by the virion from those of Vpr expressed de novo in infected cells.

### 3.4. Vpr Antagonism of HIV-1 Preintegration Silencing Is Vpr-Dose Dependent

Retroviral DNA, including that of HIV-1, is transcriptionally silenced prior to integration [33,34]. Virion-associated Vpr has been reported to counteract this silencing and stimulate expression from unintegrated HIV-1 genomes [31,32]. Therefore, our finding that Vpr-dependent enhancement of HIV-1 replication fitness in CEM.SS T cells does not require robust virion incorporation of Vpr suggests that this enhancement is not primarily due to an elevated expression of unintegrated HIV-1 genomes. To further examine this possibility, we directly assessed the impact of virion-associated Vpr on the expression of unintegrated HIV-1 in CEM.SS T cells using a dose-response experimental design.

**Figure 4:**
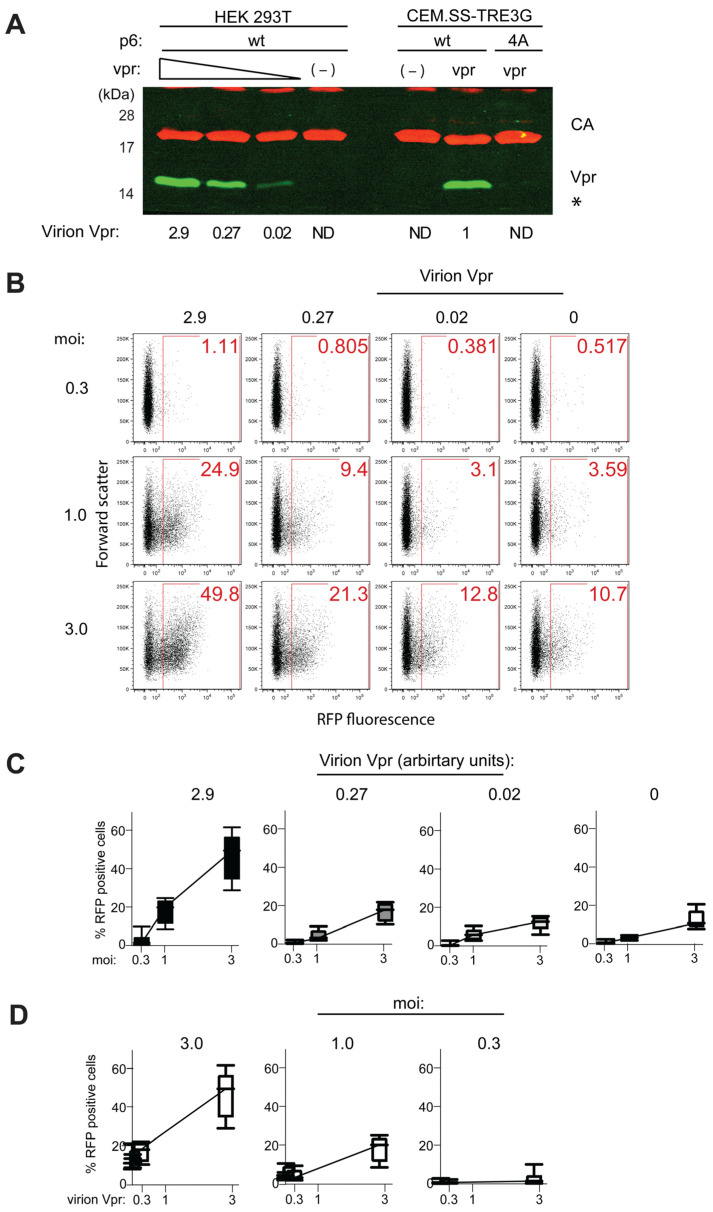
Virion-associated Vpr and expression of unintegrated HIV-1. (**A**) Levels of virion-associated Vpr in HIV-1.R-.RFP.tTA.wt produced in HEK293T cells and CEM.SS-TRE3G-vpr T cells. HIV-1 was produced in HEK293T cells co-transfected with the proviral clone and varying amounts of HIV-1 Vpr expression plasmid, or by spreading infection in CEM.SS-TRE3G-vpr T cells, in the presence of doxycycline. Relative Vpr levels were quantified as in Figure 3B and normalized to virions from CEM.SS-TRE3G-Vpr T cells. Asterisk (*) denotes a non-specific band cross-reactive with anti-Vpr antibody. (**B**) Virion associated Vpr enhances expression from unintegrated HIV-1. CEM.SS T cells cultured in the presence of 0.1 μg/mL integrase inhibitor Raltegravir were infected with the indicated four viruses produced in HEK293T cells at moi of 0.3, 1, and 3. RFP fluorescence was quantified at 48 hpi. Percentages of RFP-positive cells is shown in the upper right corner of each dot plot panel. (**C**) Box and whisker plots showing pooled results from four biological replicates of the experiment shown in (**B**). (**D**) Data used in panel (**C**) were transposed to illustrate an increase in RFP reporter expression as a function of virion-associated Vpr load, for each moi.

A panel of HIV-1 particles containing varying amounts of virion-associated Vpr was generated in HEK293T cells. These cells were co-transfected with the HIV-1.R-.RFP.tTA.wt proviral construct—bearing an inactivating Q8* mutation in the vpr gene and encoding RFP—and increasing amounts of a plasmid expressing wild-type HIV-1 Vpr. The resulting trans-complemented virions were concentrated and their lysates analyzed by immunoblotting for CA and Vpr. As shown in Figure 4A, Vpr levels in the virions spanned a more than 100-fold range, from undetectable to approximately three times higher than levels observed in virions produced from isogenic HIV-1 in CEM.SS-TRE3G-vpr T cells.

To assess the effect of virion-associated Vpr on preintegration HIV-1 expression, CEM.SS T cells were infected with virions produced in HEK293T cells at moi of 0.3, 1, or 3, in the presence of the integrase inhibitor Raltegravir. Forty-eight hpi, cells were harvested and the frequency of RFP-positive cells was measured by flow cytometry. As shown in Figure 4B,C, a dose-dependent increase in RFP-positive cells was observed, but only with virions containing the two highest levels of Vpr, and only at relatively high moi (1 or 3). This suggests that multiple infection events—and consequently, higher cumulative Vpr delivery per cell—are required to overcome silencing and activate measurable expression from unintegrated genomes. No significant effect was observed with the lowest dose of virion-associated Vpr compared to Vpr-deficient controls. Notably, even in the absence of Vpr, RFP expression from unintegrated HIV-1 genomes was not completely silenced, as evidenced by its detectability at high moi.

The low-level RFP fluorescence observed in infections carried out in the presence of an Integrase inhibitor does not reflect RFP expression from integrated proviruses resulting from rare drug-breakthrough events due to incomplete Integrase inhibition. This conclusion is supported by two observations: first, reporter viruses carrying the inactivating D116N mutation in Integrase yielded similar results (Supplementary Figure S6A); and second, the fluorescence intensity distributions were characteristic of unintegrated HIV-1, which differ markedly from those associated with integrated HIV-1 proviruses (Supplementary Figure S6B). Finally, drug-breakthrough events are unlikely to account for the observed increase in RFP expression with increasing virion-associated Vpr load at a given constant moi (Figure 4D).

Interestingly, based on the results of western blot analysis shown in Figure 4A, Vpr levels in HIV-1 particles produced from CEM.SS-TRE3G-Vpr T cells and bearing p6.wt, appear to be high enough to partially activate expression from unintegrated HIV-1. In contrast, Vpr levels in particles that were not efficiently trans-complemented either due to the presence of the p6.4A variant or an inactive tTA.** transactivator—both of which exhibit ~100-fold lower Vpr content (see Figure 3B)—appeared too low to elicit a detectable response. Collectively, these findings further support the conclusion that Vpr-mediated antagonism of preintegration silencing requires relatively high Vpr levels and is therefore unlikely to be the principal mechanism through which Vpr enhances HIV-1 replication fitness in cycling T cells.

## 4. Discussion

HIV-1 Vpr is a multifunctional accessory protein that confers a robust replication advantage to the virus in T cells. This enhancement can occur either at early post-entry stages through virion-delivered Vpr or at later stages via Vpr synthesized de novo in infected cells—or potentially both. Our previous work established an early, post-entry contribution to this effect, linked to Vpr-mediated antagonism of specific host DNA damage response enzymes. However, it also suggested that Vpr promotes HIV-1 replication via additional, as yet unidentified, mechanisms [30].

In this study, we describe a bipartite, inducible experimental system designed to disentangle the contributions of virion-associated and de novo-expressed Vpr to HIV-1 replication in T cells. This system effectively eliminates Vpr incorporation into virions while preserving its expression in infected target cells. It relies on two key components. First, the endogenous Vpr locus within the HIV-1 genome is functionally replaced by a tetracycline-dependent transcriptional transactivator (tTA), which in turn drives Vpr expression from a chromosomally integrated, tTA-responsive transgene in the target cell. This configuration enables the trans-complementation of HIV-1 particles derived from a Vpr-defective provirus with Vpr that is induced in trans after provirus integration. Second, the p6.4A mutation in the Gag protein is employed to specifically prevent virion incorporation of Vpr, without impairing viral particle release [39]. Together, these elements allow us to isolate and assess the effects of Vpr synthesized exclusively within the infected cell during the late stages of HIV-1 replication under conditions of spreading infection.

There are several mechanistic differences between how Vpr is expressed in this inducible bipartite system and how it is expressed from a native HIV-1 provirus. Under physiological conditions, Vpr is translated from a singly spliced, early HIV-1 mRNA shortly after proviral integration [53,54]. In contrast, in our bipartite system, Vpr expression follows a two-step process. In the first step, the tTA protein is expressed from a fully spliced early transcript, likely the same transcript class that normally encodes Nef, in the target cell after integration. In the second step, the de novo-expressed tTA then activates transcription of the TRE3G-vpr transgene from a tTA-inducible promoter integrated into a chromosome of the target T cell.

Although the kinetics and expression dynamics of Vpr in this system may not perfectly mirror its timing in native infection, the functional outcome—a significant enhancement of HIV-1 replication—is faithfully reproduced in the CEM.SS T cell model. Thus, we conclude that the critical biological activity of Vpr in promoting HIV-1 replication is preserved in this bipartite inducible system, and that it operates primarily through mechanisms that do not require its presence in the incoming virion.

HIV-1 virions with wild-type p6 produced from T cells were reported to contain several hundred copies of Vpr [55]. In contrast, in HIV-1 virions with the p6.4A mutation trans-complemented with Vpr in CEM.SS_TRE3G-Vpr T cells, the copy number was reduced by more than 100-fold. Thus, in the presence of p6.4A, Vpr incorporation into virions is effectively abolished. Remarkably, our data show that the p6.4A mutation has no measurable impact on viral replication fitness, even when assessed using sensitive pairwise competition assays in CEM.SS T cells. We therefore conclude that this p6.4A mutation functionally separates the replication-supporting activities of the virion-associated Vpr at early post-entry stages from those of the de novo synthesized Vpr during the late stages of the HIV-1 life cycle.

The ability of the p6.4A mutation to decouple these functions allowed us to directly test whether Vpr acts post-integration to enhance HIV-1 replicative fitness. Remarkably, using pairwise replication competition assays, no significant differences in replication kinetics were observed between viruses encoding wild-type p6 versus p6.4A in the presence or absence of Vpr. This indicates that robust virion incorporation of Vpr is not required for its effect on viral fitness. Furthermore, Vpr enhanced HIV-1 replication to a similar extent regardless of whether both competing viruses carried wild-type or mutant (4A) p6 sequences. Together, these results demonstrate that virion-associated Vpr is dispensable for replication enhancement in dividing T cells and strongly support the conclusion that this activity is mediated by Vpr de novo expressed in the infected cell, after integration.

Although the precise mechanism by which Vpr enhances HIV-1 replication post-integration remains to be fully defined, existing evidence links to Vpr interactions with the transcription control machineries. Notably, HIV-1 expression is known to be optimal in the G2 phase of the cell cycle [26], and the effect of Vpr on HIV-1 replication fitness in T cells depends entirely on Vpr’s capacity to induce cell cycle arrest in the G2 phase and to hijack CRL4^DCAF1^ E3 ubiquitin ligase complex [30]. Vpr interaction with this E3 enzyme is required for the induction of the G2 arrest. By hijacking this ligase through binding to its DCAF1 substrate receptor [41,56,57], Vpr redirects CRL4^DCAF1^ to target a degradation specific set of host proteins that would otherwise impair HIV-1 replication. This set includes several key players in transcriptional and epigenetic repression pathways including the SMC5/6 complex, TET2, CCDC137, CTIP2, and various histone deacetylases (HDACs) [31,37,58,59]. Other targets antagonized by Vpr via CRL4^DCAF1^—such as HLTF, Exo1, SIRT7, PU.1—and additional, yet unidentified proteins may also contribute [29,30,52,60]. Teasing apart the contributions of these various targets may be challenging due to potential combinatorial effects and the limited sensitivity of current tools.

Our inability to detect a robust component conferred by the virion-associated Vpr is somewhat surprising, given that such Vpr promotes the expression of the incoming unintegrated HIV-1 genomes [32]. This activity is mediated, at least in part, via CRL4^DCAF1^-dependent antagonism of SLF2 and the SMC5/6 complex [31]. Indeed, as shown here, virion-associated Vpr can induce detectable reporter gene expression from unintegrated viral DNA in CEM.SS T cells. However, several factors may explain the relatively minor role of this activity in promoting replication fitness in our system.

First, evidence suggests that HIV-1 may not have evolved to maximize virion-associated Vpr activity. In particular, Vpr is incorporated into virions with low efficiency; the Vpr:p6 stoichiometry is substoichiometric by approximately an order of magnitude [55]. In contrast, Vpr levels in virions generated in HEK293T cells by trans-complementation are far higher due to transient overexpression of Vpr in this system. Significantly, our dose-response experiments show that the magnitude of Vpr-induced gene expression from unintegrated HIV-1 DNA is strongly dose-dependent. Moreover, the unintegrated HIV-1 DNA does not appear to be completely repressed even in the absence of Vpr. This may explain why virion-associated Vpr has only a minor effect on HIV-1 replication in proliferating highly transcriptionally active cells, in which the dominant effect of Vpr on replication fitness arises post-integration. Second, it is also conceivable that virion packaged Vpr plays a more prominent role in non-dividing resting T cells, which may more actively repress the incoming DNA [61,62] and/or in other cell types such as macrophages and dendritic cells.

Third, a compelling alternative hypothesis is that Vpr-mediated antagonism of host transcriptional repressors—such as SMC5/6, HDACs, and/or CCDC137 and others—plays a critical role not before but after provirus integration, by preventing epigenetic silencing of the already integrated HIV-1 genome. This concept is supported by precedent from Vpx, an accessory protein of HIV-2 and several simian immunodeficiency viruses (SIVs), which is related to Vpr [63,64]. Vpx antagonizes HUSH complex, a repressor of integrated proviral transcription, also by hijacking CRL4^DCAF1^ [65]. Like Vpr, Vpx is packaged into virions and delivered to the target cell upon entry [66,67]. Critically, Vpx-mediated HUSH antagonism can reactivate latent, silenced proviruses, suggesting that this activity, exerted post-integration, may help ensure the productive establishment of infection in newly infected cells [65,68]. By analogy, it is plausible that Vpr acts post-integration to relieve epigenetic repression and facilitate transcriptional activity of the integrated provirus—a function potentially essential for efficient viral replication and transmission in T cells.

## Data Availability

The raw data supporting the conclusions of this article will be made available by the authors on request.

## Supplementary Materials

The following supporting information can be downloaded at https://www.mdpi.com/article/10.3390/v17070958/s1, Figure S1: Nucleotide sequence of the mRFP-2A-tTA.wt-IRES-nef cassette and peptides it encodes. Figure S2: Unprocessed original image of western blot in Figure 2C. Figure S3: Unprocessed original image of western blot in Figure 2D. Figure S4: Unprocessed original image of western blot in Figure 3B. Figure S5: Unprocessed original image of western blot in Figure 4A. Figure S6: Expression from unintegrated HIV-1: effects of the Integrase D116N substitution and RFP fluorescence intensity profiles.
