# Supplementary material for: De Novo Expressed Vpr Stimulates HIV-1 Replication in T Cells
Source: Viruses. 2025 Jul 7;17(7):958. doi: 10.3390/v17070958 (PMC12297976; doi:10.3390/v17070958)
Supplement: Supplementary file 1 [file viruses-17-00958-s001.zip › viruses-3730049-supplementary.pdf]

Supplementary Materials

Figure S1: Nucleotide sequence of the mRFP-2A-tTA.wt-IRES-nef cassette and peptides it encodes.

Figure S2: Unprocessed original image of western blot in Figure 2C

Figure S3: Unprocessed original image of western blot in Figure 2D

Figure S4: Unprocessed original image of western blot in Figure 3B

Figure S5: Unprocessed original image of western blot in Figure 4A

Figure S6: Expression from unintegrated HIV-1: effects of the Integrase D116N substitution and RFP fluorescence intensity profiles.

Nef-mRFP-A2-tTA-IRES-Nef-XhoI cassette

atg ggt ggc g**cg gcc g**ca gtg tct aag ggc gaa gag ctg att aag gag aac atg cac atg aag ctg tac atg gag
M G G A A A V S K G E E L I K E N M H M K L Y M E 25

ggc acc gtg aac aac cac cac ttc aag tgc aca tcc gag ggc gaa ggc aag ccc tac gag ggc acc cag acc atg
G T V N N H H F K C T S E G E G K P Y E G T Q T M 50

aga atc aag gtg gtc gag ggc ggc cct ctc ccc ttc gcc ttc gac atc ctg gcc aca tct ttt atg tat ggg tct
R I K V V E G G P L P F A F D I L A T S F M Y G S 75

cgc aca ttc atc aac cac acc cag ggc atc ccc gac ttc ttt aag cag tcc ttc cct gag ggc ttc aca tgg gag
R T F I N H T Q G I P D F F K Q S F P E G F T W E 100

aga gtc acc aca tac gaa gac ggg ggc gtg ctg acc gct acc cag gac acc agc ctc cag gac ggc tgc ctc atc
R V T T Y E D G G V L T A T Q D T S L Q D G C L I 125

tac aac gtc aag atc aga ggg gtg aac ttc cca tcc aac ggc cct gtg atg cag aag aaa aca ctc ggc tgg gag
Y N V K I R G V N F P S N G P V M Q K K T L G W E 150

gcc aac acc gag atg ctg tac ccc gct gac ggc ggc ctg gaa ggc aga acc gac atg gcc ctg aag ctc gtg ggc
A N T E M L Y P A D G G L E G R T D M A L K L V G 175

ggg ggc cac ctg atc tgc aac ttc aag acc aca tac aga tcc aag aaa ccc gct aag aac ctc aag atg ccc ggc
G G H L I C N F K T T Y R S K K P A K N L K M P G 200

gtc tac tat gtg gac cac aga ctg gaa aga atc aag gag gcc gac aaa gag acc tac gtc gag cag cac gag gtg
V Y Y V D H R L E R I K E A D K E T Y V E Q H E V 225

gct gtg gcc aga tac tgc gac ctc cct agc aaa ctg ggg cac aaa ctt aat ggc gga gga acg cgt ggc agt gga
A V A R Y C D L P S K L G H K L N G G G T R G S G 250

gag ggc aga gga agt ctg cta aca tgc ggt gac gtc gag gag aat cct ggc cca gtc gac atg gat **tca aga** ctg
E G R G S L L T C G D V E E N P G P V D M D S R L 275

gac aag agc aaa gtc ata aac gga gct ctg gaa tta ctc aat ggt gtc ggt atc gaa ggc ctg acg aca agg aaa
D K S K V I N G A L E L L N G V G I E G L T T R K 300

ctc gct caa aag ctg gga gtt gag cag cct acc ctg tac tgg cac gtg aag aac aag cgg gcc ctg ctc gat gcc
L A Q K L G V E Q P T L Y W H V K N K R A L L D A 325

ctg cca atc gag atg ctg gac agg cat cat acc cac ttc tgc ccc ctg gaa ggc gag tca tgg **caa** gac ttt ctg
L P I E M L D R H H T H F C P L E G E S W Q D F L 350

cgg aac aac gcc aag tca tac cgc tgt gct ctc ctc tca cat cgc gac ggg gct aaa gtg cat ctc ggc acc cgc
R N N A K S Y R C A L L S H R D G A K V H L G T R 375

cca aca gag aaa cag tac gaa acc ctg gaa aat cag ctc gcg ttc ctg tgt cag caa ggc ttc tcc ctg gag aac
P T E K Q Y E T L E N Q L A F L C Q Q G F S L E N 400

gca ctg tac gct ctg tcc gcc gtg ggc cac ttt aca ctg ggc tgc gta ttg gag gaa cag gag cat caa gta gca
A L Y A L S A V G H F T L G C V L E E Q E H Q V A 425

aaa gag gaa aga gag aca cct acc acc gat tct atg ccc cca ctt ctg aga caa gca att gag ctg ttc gac cgg
K E E R E T P T T D S M P P L L R Q A I E L F D R 450

cag gga gcc gaa cct gcc ttc ctt ttc ggc ctg gaa cta atc ata tgt ggc ctg gag aaa cag cta aag tgc gaa
Q G A E P A F L F G L E L I I C G L E K Q L K C E 475

agc ggc ggg ccg acc gac gcc ctt gac gat ttt gac tta gac atg ctc cca gcc gat gcc ctt gac gac ttt gac
S G G P T D A L D D F D L D M L P A D A L D D F D 500

ctt gat atg ctg cct gct gac gct ctt gac gat ttt gac ctt gac atg ctc ccc ggg tga gtcgac cccccctaacgtt
L D M L P A D A L D D F D L D M L P G * 529

actggccgaagccgcttggaataaggccggtgtgcgtttgtctatatgttattttccaccatattgccgtcttttggcaatgtgagggcccggaaacct

ggccctgtcttcttgacgagcattcctaggggtctttcccctctcgccaaaggaatgcaaggtctgttgaatgtcgtgaaggaagcagttcctctggaa

gcttcttgaagacaaacaacgtctgtagcgaccctttgcaggcagcggaaccccccacctggcgacaggtgcctctgcggccaaaagccacgtgtataa

gatacacctgcaaaggcggcacaaccccagtgccacgttgtgagttggatagttgtggaaagagtcaaatggctctcctcaagcgtattcaacaagggg

ctgaaggatgcccagaaggtaccccattgtatgggatctgatctggggcctcggtacacatgctttacatgtgtttagtcgaggttaaaaaaacgtcta

ggccccccgaaccacggggacgtggttttcctttgaaaaacacgatgataat atg ggt ggc aag tgg tca aaa agt agt gtg att gga
M G G K W S K S S V I G 12

tgg cct gct gta agg gaa aga atg aga cga gct gag cca gca gca gat ggg gtg gga gca gta t**ct cga g**
W P A V R E R M R R A E P A A D G V G A V S R 35

```
atgggtggcgcggccgcagtgtctaagggcgaagagctgattaaggagaacatgcacatgaagctgtacatggagggcaccgtgaacaaccaccacttcaagtgcacatcc
gagggcgaaggcaagccctacgagggcacccagaccatgagaatcaaggtggtcgagggcggccctctcccccttcgccttcgacatcctggccacatcttttatgtatggg
tctcgcacattcatcaaccacacccagggcatccccgacttctttaagcagtccttccctgagggcttcacatgggagagagtcaccacatacgaagacgggggcgtgctg
accgctacccaggacaccagcctccaggacggctgcctcatctacaacgtcaagatcagaggggtgaacttcccatccaacggccctgtgatgcagaagaaaacactcggc
tgggaggccaacaccgagatgctgtaccccgctgacggcggcctggaaggcagaaccgacatggccctgaagctcgtgggcgggggccacctgatctgcaacttcaagacc
acatacagatccaagaaacccgctaagaacctcaagatgcccggcgtctactatgtggaccacagactggaaagaatcaaggaggccgacaaagagacctacgtcgagcag
cacgaggtggctgtggccagatactgcgacctccctagcaaactggggcacaaacttaatggcggaggaacgcgtggcagtggagagggcagaggaagtctgctaacatgc
ggtgacgtcgaggagaatcctggcccagtcgacatggattcaagactggacaagagcaaagtcataaacggagctctggaattactcaatggtgtcggtatcgaaggcctg
acgacaaggaaactcgctcaaaagctgggagttgagcagcctaccctgtactggcacgtgaagaacaagcgggccctgctcgatgccctgccaatcgagatgctggacagg
catcatacccacttctgcccccctggaaggcgagtcatggcaagactttctgcggaacaacgccaagtcataccgctgtgctctcctctcacatcgcgacggggctaaagtg
catctcggcacccgcccaacagagaaacagtacgaaaccctggaaaatcagctcgcgttcctgtgtcagcaaggcttctccctggagaacgcactgtacgctctgtccgcc
gtgggccactttacactgggctgcgtattggaggaacaggagcatcaagtagcaaaagaggaaagagagacacctaccaccgattctatgcccccacttctgagacaagca
attgagctgttcgaccggcagggagccgaacctgccttccttttcggcctggaactaatcatatgtggcctggagaaacagctaaagtgcgaaagcggcgggccgaccgac
gcccttgacgattttgacttagacatgctcccagccgatgcccttgacgactttgaccttgatatgctgcctgctgacgctcttgacgattttgaccttgacatgctcccc
gggtgagtcgacccccctaacgttactggccgaagccgcttggaataaggccggtgtgcgtttgtctatatgttattttccaccatattgccgtcttttggcaatgtgagg
gcccggaaacctggccctgtcttcttgacgagcattcctaggggtctttcccctctcgccaaaggaatgcaaggtctgttgaatgtcgtgaaggaagcagttcctctggaa
gcttcttgaagacaaacaacgtctgtagcgaccctttgcaggcagcggaaccccccacctggcgacaggtgcctctgcggccaaaagccacgtgtataagatacacctgca
aaggcggcacaaccccagtgccacgttgtgagttggatagttgtggaaagagtcaaatggctctcctcaagcgtattcaacaaggggctgaaggatgcccagaaggtaccc
cattgtatgggatctgatctggggcctcggtacacatgctttacatgtgtttagtcgaggttaaaaaaacgtctaggccccccgaaccacggggacgtggttttcctttga
aaaacacgatgataatatgggtggcaagtggtcaaaaagtagtgtgattggatggcctgctgtaagggaaagaatgagacgagctgagccagcagcagatggggtgggagc
agtatctcgag
```

Figure S1. Nucleotide sequence of the mRFP-2A-tTA.wt-IRES-nef cassette and the peptides it encodes.

The EagI and XhoI recognition sequences are underlined and shown in bold. Coding sequences are color-coded as follows: Nef in green, mRFP in magenta, 2A in blue, tTA in yellow, and the IRES element in gray. Peptide spacers are not highlighted. tTA codons for S3, R4, and Q77 (corresponding to S273, R274, and Q347 in the polyprotein, respectively) that are mutated to stop codons in the tTA.** variant are shown in bold red. Numbers on the left indicate the position of the leftmost amino acid in the polypeptide sequence. mRFP translation initiates from the native nef M1 start codon.

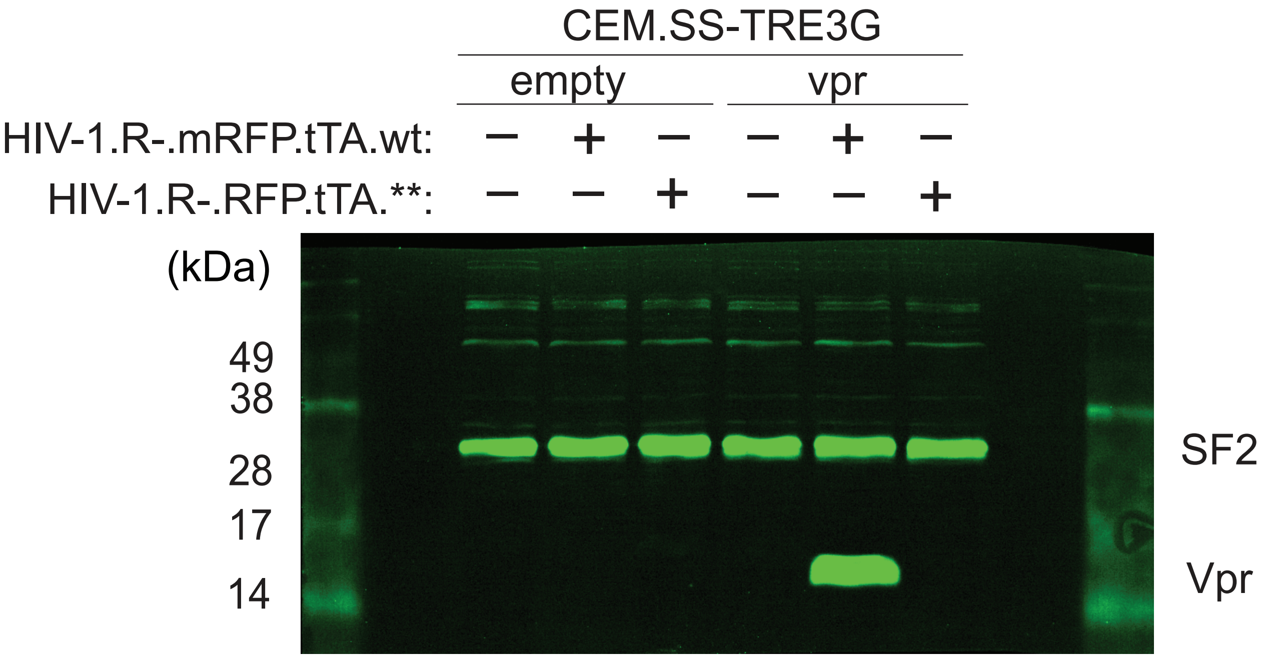

Figure S2: Unprocessed original image of western blot shown in Figure 2C

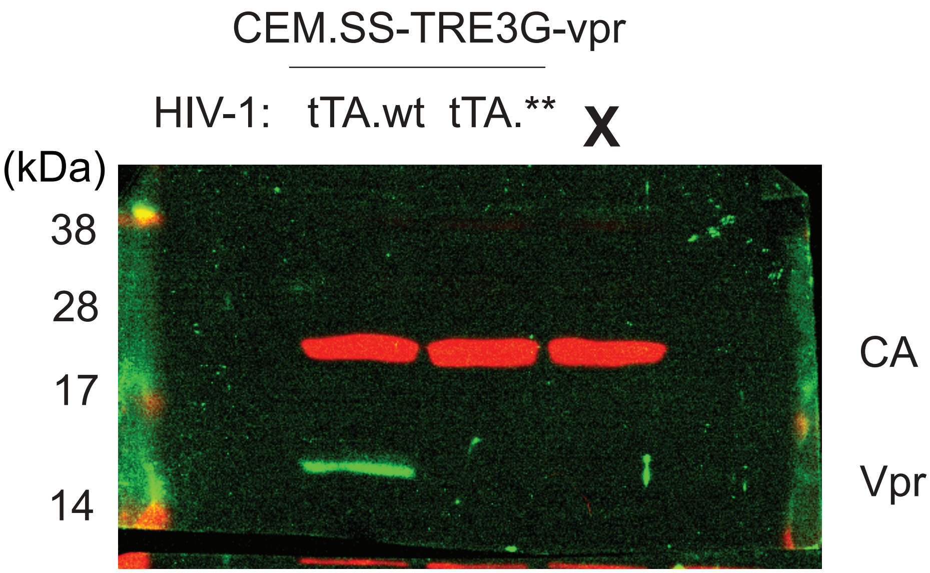

Figure S3: Unprocessed original image of western blot shown in Figure 2D

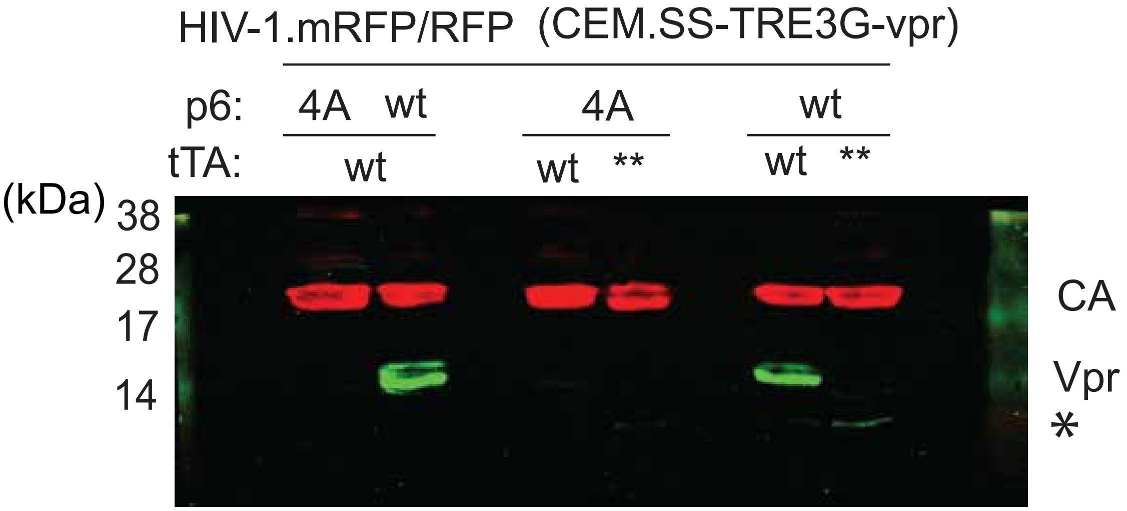

Figure S4: Unprocessed original image of western blot shown in Figure 3B

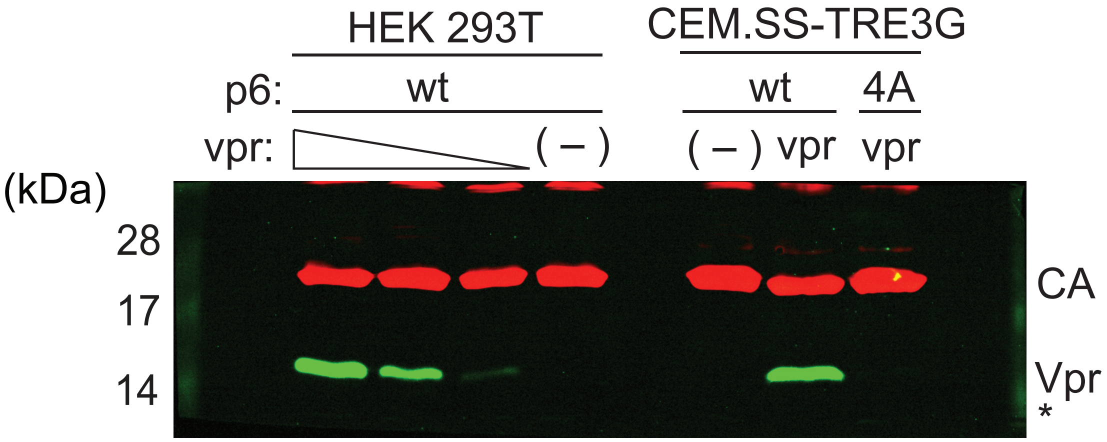

Figure S5: Unprocessed original image of western blot shown in Figure 4A

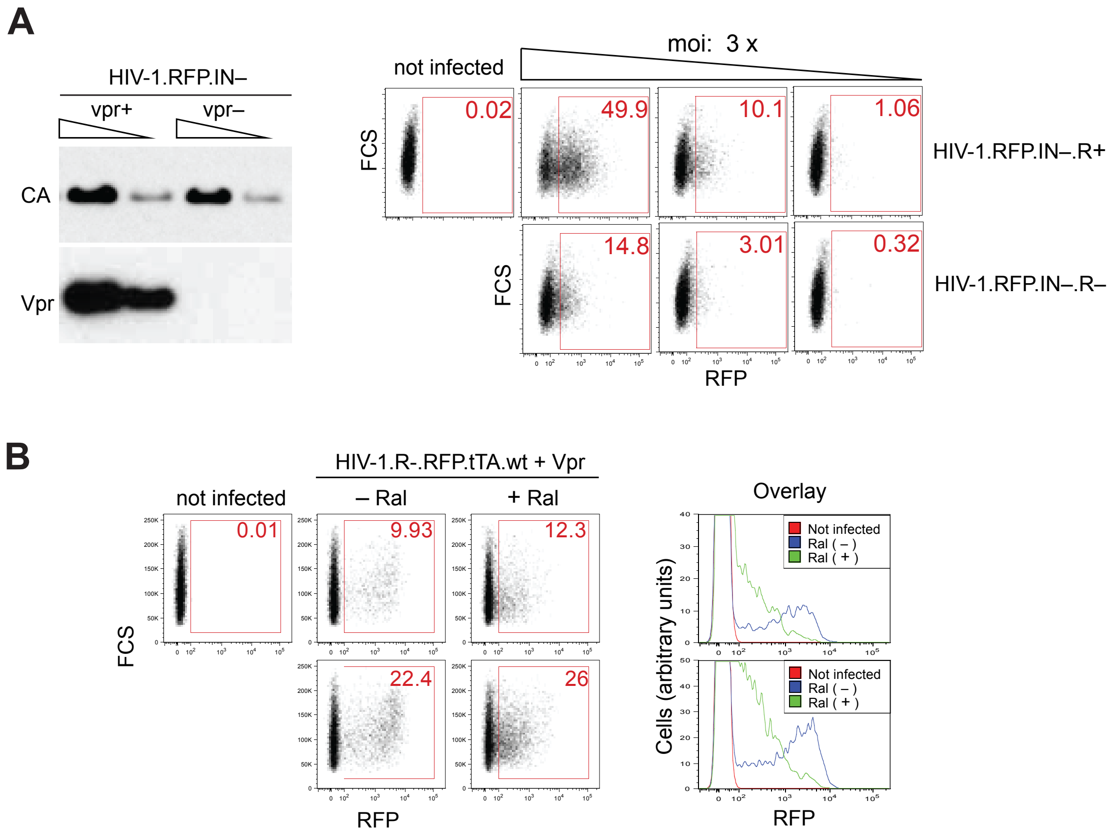

Figure S6: Expression from unintegrated HIV-1: effects of the Integrase D116N substitution and RFP fluorescence intensity profiles.

A. Virion associated Vpr enhances expression from HIV-1 carrying the inactivating D116N Integrase substitution.
CEM.SS T cells were infected with 3-fold serial dilutions of HIV-1 reporter viruses carrying the inactivating IN.D116N substitution. The viruses were isogenic except for a point mutation inactivating the vpr gene in one of the viruses (R+, R–). Left panel: Two-fold dilutions of the normalized viruses were analyzed by immunoblotting for CA and Vpr, as indicated. Right panel: Dot plots illustrating RFP fluorescence profiles of infected cells at 3 dpi, recorded using an LSRFortessa flow cytometer. The percentage of RFP-positive cells is indicated in the upper right corner of each plot.

B. RFP fluorescence intensity profiles differ between integrated and unintegrated HIV-1.
CEM.SS T cells were infected with a vpr-defective HIV-1 reporter virus transcomplemented with Vpr and expressing an RFP marker, in the absence or presence of Raltegravir, across a wide range of mois. Dot plots showing comparable percentages of RFP-positive cells resulting from infections performed with or without Raltegravir were selected for comparison: upper panels, ~10%; lower panels, ~20%. Overlays of RFP fluorescence intensity histograms for each corresponding set are shown in the right panels
